# Laparoscopic Reversal of Gastric Bypass: A Retrospective Review

**DOI:** 10.1007/s11695-025-08269-7

**Published:** 2025-09-23

**Authors:** Ahmed Abdelsalam, Ahmed Ghobashy, Ramy Elhawary, Michael Shenouda, Ahmed Khaled, Ahmed Abdellatif

**Affiliations:** Cairo University, Giza, Egypt

**Keywords:** Bypass reversal, One anastomosis gastric bypass, Roux-en-Y gastric bypass

## Abstract

**Background:**

Gastric bypass can result in serious complications such as dumping syndrome, Malnutrition, and chronic abdominal pain refractory to symptomatic treatment and necessitate surgical intervention in the form of reversal to normal anatomy. Our study draws on a 5-year experience in the reversal of gastric bypass, addressing indications, operative techniques, and complications associated with this procedure.

**Methods:**

This retrospective analysis pilot study included ten patients who underwent reversal of either Roux-en-Y gastric bypass (RYGB) or one-anastomosis gastric bypass (OAGB) and investigated their indications, complications, and outcomes.

**Results:**

Ten patients underwent gastric bypass reversal; 60% of them had a reversal of OAGB, while 40% had a reversal of RYGB. The main indications for bypass reversal were malnutrition (hypoalbuminemia) (33%), excessive loss of weight (29%), followed by chronic abdominal pain, chronic anemia, diarrhea, non-healing Marginal ulcer, and persistent reflux, representing 10% each. Follow-up was achieved in 90% of patients at 180 days (6 months), and the overall postoperative morbidity was 10%. Within the 6 months, there was a single mortality event (10%) attributed to preexisting liver cell failure. The mean BMI preoperatively and postoperatively were 26.2 kg/m^2^ and 27.9 kg/m^2^, respectively (*p*-value = 0.013) at 6 months, while the mean serum albumin levels preoperatively and postoperatively were 2.8 g/dl and 3.4 g/dl, respectively (*p*-value = 0.019).

**Conclusions:**

Laparoscopic reversal of gastric bypass is a complex surgery requiring a specialized surgical center, and it should be a last resort for intractable chronic symptoms. Patient education about relatively high morbidity and the possibility of dissatisfaction is crucial.

## Introduction

Gastric bypass surgeries are commonly performed as metabolic bariatric surgeries [1]. The anatomic changes of gastric bypass allow the food bolus to reach the jejunum directly through the gastrojejunostomy, and the excluded stomach and duodenum have no contact with the ingested food bolus. These anatomic changes lead to alterations in glucose kinetics, absorption of minerals and micronutrients [2], and postprandial levels of gastrointestinal (GI) and pancreatic hormones [3]. With dietary adjustments and supplementation of standard micronutrients and minerals, most patients experience benefits from these changes after undergoing gastric bypass. Although primary metabolic surgery typically has low instances of early postoperative complications such as bleeding or leaks from anastomosis/staple lines, there may be long-term issues that may develop. After gastric bypass surgery, these complications may encompass what is known as “dumping” syndrome, reactive hyperinsulinemia leading to hypoglycemia [4], significant weight loss or weight regain [5], marginal ulcer formation [6], and malabsorption of vitamins or minerals that can cause anemia or ongoing abdominal pain, which can complicate effective symptomatic management [7]. Indications for reversal are primarily in cases that are unresponsive to conservative, multidisciplinary Management. For Malnutrition, the patient cannot sustain his basic nutritional requirements through oral intake, as evidenced by massive weight loss and persistent hypoalbuminemia. There is no clear definition of what massive weight loss is, but generally, when BMI is less than 18 kg/m^2^ or the patient is dissatisfied with their weight loss, it is considered an indication for reversal. For marginal ulcers, when all medical regimens, such as proton pump inhibitors, fail, including failure of endoscopic management, surgical intervention may be necessary. For dumping syndrome, when all dietary modifications and medical treatments, such as acarbose, diazoxide, and octreotide, fail to prevent hypoglycemic attacks, further evaluation and treatment options should be considered [8]. Surgical interventions—classified as revisional, conversional, or reversal procedures aimed at restoring the original pre-bariatric anatomy—may become necessary [9].

Reversal of the gastric bypass to normal anatomy is recommended for patients experiencing rare, severe chronic complications. In this study, we report our extensive, single-center experience with the laparoscopic reversal of gastric bypass procedures over a 5-year period. The paucity of patients in prior studies has resulted in limited knowledge regarding the indications and complications.

This study aims to provide a deeper understanding of the most common indications, perioperative management strategies, and clinical outcomes in patients who undergo gastric bypass reversal at a specialized bariatric center.

## Patients and Methods

A retrospective review was conducted involving patients who underwent gastric bypass reversal at the General and Laparoscopic Surgery Department of a tertiary care university-affiliated hospital between April 2020 and April 2025. It included patients aged between 18 and 70 who underwent reversal of gastric bypass procedures, namely Roux-en-Y gastric bypass and one-anastomosis gastric bypass. We excluded patients who had undergone conversion to another bariatric procedure, such as sleeve gastrectomy, or who had a history of previous intestinal surgery.

### Sample Size

The sample size was calculated using the EPI INFO sample size calculator for analytic studies, with a 0.05 alpha error and a power of the study of 0.80 and confidence interval of 95%. According to literature, the indications of reversal of gastric bypass were malnutrition or excessive weight loss (31.5% of patients) and chronic abdominal pain (26.3%). The outcome and complications: 26.3% of patients required additional delayed procedures, and in 10%, the diarrhea persisted for 7 days or longer. The sample size calculated for studying the indications, outcomes, and complications of gastric bypass reversal was ten patients. Sampling technique: A convenient sample of patients with the inclusion and exclusion criteria was assigned to the study until the total sample size calculated was reached.

### Ethical Consideration

The study protocol received approval from the Institutional Ethical Research Committee. All participants were fully informed about the study’s purpose and the procedure involved. Written informed consent was obtained prior to enrollment, along with a detailed explanation of the potential benefits and risks associated with the study. In compliance with the Declaration of Helsinki, all data collection, entry, and analysis processes were carried out with strict attention to confidentiality and privacy.

### Preoperative Assessment

Detailed history taking was conducted regarding anthropometric measures (body mass index), date and type of the primary bariatric surgery performed, current symptoms, and their severity.

We used a questionnaire survey created by a Danish study published in 2024 to assess the severity of symptoms and self-assessed sensation of well-being [5].

Patients completed the questionnaire survey form both preoperatively and 6 months postoperatively. The questionnaire gathered data on patients’ height, weight, employment status, and the presence of complications or symptoms both before gastric bypass reversal (pre-reversal) and after the procedure (post-reversal). To assess symptom severity, patients were asked whether their symptoms had prompted them to seek medical attention. A symptom severity scoring system was used, assigning: 1 point for no healthcare contact, 2 points for contact with a general practitioner, 3 points for visits to an outpatient specialist clinic, and 4 points for hospital admission. This score was recorded both preoperatively and at 6 months postoperatively.

After detailed history taking and questionnaire survey filling, we performed a laboratory check-up on nutritional status (serum Albumin), anemia (hemoglobin), minerals, and vitamin panel (vitamins A, B, C, D, and E, zinc, iron) to check for severe deficiencies.

A gastric volumetry CT scan was performed preoperatively. The device used was a Toshiba Aquilin® 16-slice, 7.5 MHU X-ray Tube CT scanner with dual 18-inch flat-panel LCD monitors. Gastrografin was used as an oral contrast to delineate the gastrointestinal tract (GIT), pouch size, and presence/absence of gastro-gastric fistula.

The decision to proceed with laparoscopic reversal of gastric bypass was made by a multidisciplinary team (MDT) comprising surgeons, dietitians, and endocrinologists. Each patient underwent a thorough evaluation based on their specific complications or symptoms, and all appropriate medical and dietary treatments were offered beforehand, for example, somatostatin analogues in cases of severe dumping syndrome.

#### Surgical Technique for Reversal of Gastric Bypass

For laparoscopic reversal (undo) of Roux-en-Y gastric bypass (RYGB), patients were positioned supine with leg-compression devices. General anesthesia was induced, together with antibiotic prophylaxis. A five-port technique, similar to that used for primary RYGB, was employed, including a 5 mm liver retractor inserted via a subxiphoid stab incision. The Roux limb, biliopancreatic (BP) limb, and common channel (CC) were carefully identified. The lesser sac was accessed behind the gastric pouch and Roux limb, which was then detached from the pouch and repositioned into the lower abdomen. Restoration of gastric continuity was achieved by re-anastomosing the excluded stomach and gastric pouch using an endo-stapler, followed by closure of the gastrotomies in two layers.

If the Roux limb was to be removed, the decision was based on intraoperative assessment of adhesion severity and vascular compromise. It was dissected from the jejunojejunostomy without narrowing the anastomosis and extracted through a 12 mm port. Alternatively, if the Roux limb was to be brought back into continuity, the BP limb was divided from the jejunojejunostomy with an endo-stapler, again preserving anastomotic width. Enterotomies were created in the distal BP limb and proximal Roux limb to introduce and fire an endo-stapler, forming a new anastomosis; the common enterostomy was then closed in a single layer.

For laparoscopic reversal of one-anastomosis gastric bypass (OAGB), normal anatomy was restored, first by dismantling the prior gastrojejunostomy (GJ) to separate the jejunum from the pouch without resection. Then, gastro-gastric (GG) anastomosis was created with an endo-stapler, and sometimes two linear endostaplers were used for long pouches to ensure proper function of the stomach and avoid any risk of restriction. Drain placement was per the surgeon’s preference, and skin closure was performed before emerging from anesthesia. Postoperative diet progression mirrored that of primary bariatric procedures.

#### Postoperative Follow-Up

Patients were followed postoperatively at 1 week and then at scheduled intervals. Clinical outcomes included resolution of symptoms, weight regain, nutritional status based on labs, and postoperative complications.

#### Statistical Methods

Data entry was performed using SPSS (Statistical Package for the Social Sciences) version 27.0 (IBM, SPSS, USA). Categorical variables were presented in count and percentage, and quantitative variables were presented in mean, standard deviation, minimum, and maximum. Any *p*-value < 0.05 was considered significant.

## Results

### Patients’ Characteristics

Out of 504 patients who underwent RYGB or OAGB in our institution, ten patients (*N* = 10) underwent reversal of gastric bypass (1.98%). Eight patients were females, and two males were included in this study. The mean age (± SD) was 39.6 (± 9.8) years.

The routine preoperative psychological evaluation for all patients revealed no psychological contradiction for reversal procedures. No comorbidities were found in any of our patients except for nutritional liver cell failure in one patient due to previous OAGB.

Six patients (60%) underwent a reversal of OAGB, while four patients (40%) had a reversal of RYGB (Table 1).

**Table 1:** Previous surgeries and indications of reversal

	Frequency	Percent
Previous surgeries		
OAGB	6	60%
RYGB	4	40%
Cause of reversal		
Malnutrition (hypoalbuminemia)	3	30%
Weight loss	2	20%
Chronic abdominal pain	1	10%
Chronic anemia	1	10%
Diarrhea and poor lifestyle	1	10%
Non healing marginal ulcer	1	10%
Persistent reflux after RYGB	1	10%

The mean time from gastric bypass to its reversal was 27.4 (± 15.6) months, ranging from 9 to 50 months. The mean (± SD) operative time of reversal surgery was 65.0 (± 10.0) min, with a range of 50 to 80 min.

The mean biliopancreatic limb length was 91 (± 8) cm in cases of RYGB. While in OAGB, the gastrojejunostomy was at a mean distance of 190 (± 19) cm from the DJ.

The main indications for reversal were malnutrition (persistent hypoalbuminemia) (33%) and excessive weight loss (29%) followed by chronic abdominal pain, chronic anemia, diarrhea, poor lifestyle, non-healing Marginal ulcer, and persistent reflux after RYGB, representing 10% each (Table 1).

The rate of follow-up at 180 days (6 months) was 90%, and the overall postoperative morbidity rate within the 6 months was 10% (leakage). This case underwent reversal of RYGB due to chronic abdominal pain. On postoperative day 5, the patient was vitally unstable with repeated vomiting. After resuscitation, a CT scan was done, which revealed leakage from the anastomotic line between the distal biliopancreatic limb and the proximal Roux limb, which was taken down and re-anastomosed. The patient experienced a smooth postoperative recovery and was discharged uneventfully 6 days later.

There was a single death attributed to liver cell failure (10%). This patient has undergone OAGB 3 years prior to the presentation and suffered from severe malnutrition and liver cell failure. The patient received supportive treatment in the form of IV fluids, albumin, vitamins (Thiamin, B12), electrolyte correction, and TPN. The patient was discharged after 1 week for a home regimen of TPN. After the MDT consultation and the decision to reverse was Made, the patient was admitted, and supportive treatment was initiated to improve his overall condition. After optimization of the general condition confirmed by preoperative labs and clinical assessment, reversal was done after 5 days. Still, unfortunately, due to nutritional liver cell failure, the patient developed generalized anasarca with hepatic encephalopathy and died.

A sensitivity analysis excluding the lost-to-follow-up patient did not alter the significance of primary outcomes.

The analysis of symptom severity scores showed significant improvement following laparoscopic gastric bypass reversal. Preoperative scores ranged from 2 to 4 (median 3, IQR 2–3), declining postoperatively to 1–3 (median 1.5, IQR 1–2), with a *p*-value of 0.024. Notable reductions were observed in malnutrition (e.g., case of Malnutrition improved from 3 to 1), excessive weight loss (2 to 1), and persistent reflux (3 to 1). Chronic abdominal pain and diarrhea scores remained unchanged (2 to 2 and 3 to 3, respectively). Non-healing Marginal ulcers improved from 4 to 2, aligning with metabolic benefits reflected in albumin normalization and restoration of the normal anatomy.

Regarding quality of life at 6 months postoperatively, 75% of patients reported that the reversal surgery had improved their quality of life (Table 2) according to the questionnaire created by the Danish Study in 2024.

**Table 2:** Quality of life at 6-month follow-up among participating patients (*n* = 8)

Quality of life	Frequency	Percent
Much better	4	50.0
Better	2	25.0
Same	1	12.5
Worse (reflux)	1	12.5
Total*	8	100.0

^*^One patient lost follow-up, one patient died

Approximately two-thirds of the patients (62.5%) regained weight at 6 months after the reversal surgery. The mean BMI increased from 25.4 (± 3.2) kg/m^2^ to 27.9 (± 2.1) kg/m^2^, a statistically significant change with a *p*-value of 0.013 (Table 3). While statistically significant, the mean BMI increase of 1.5 kg/m^2^ may not reflect substantial clinical weight regain.

**Table 3:** Statistical difference in BMI and albumin between preoperative and postoperative among participating patients (*n* = 8)

	Mean	Standard deviation	*p*-value	Effect size
BMI				
Preoperative	25.4	3.2	0.013	2.26
6 m postoperative	27.9	2.1		
Serum albumin g/dl				
Preoperative	2.95	0.59	0.019	0.45
6 m postoperative	3.40	0.24		

The mean (± SD) of albumin preoperatively and postoperatively was 2.95 (± 0.59) g/dl/and 3.4 (± 0.24) g/dl, respectively, with a *p*-value of 0.019 (Table 3).

## Discussion

In the current study, the primary indications for reversal were malnutrition (hypoalbuminemia, 33%) and excessive weight loss (29%), followed by chronic abdominal pain, chronic anemia, and other complications (10% each). These findings are consistent with prior studies, such as the study by Himpens et al. [1], which identified malnutrition and intractable symptoms as key reasons for reversal [1]. Similarly, a Danish study (2024) reported malnutrition and excessive weight loss as leading causes, though their cohort had a higher proportion of patients with chronic abdominal pain (26.3%) [5]. This discrepancy may reflect differences in patient selection or preoperative management strategies.

The mean operative time in our study was 65 min, which is comparable to other series reporting laparoscopic reversal times ranging from 60 to 120 min. Svensson et al. [4] reported a mean operative time of 70–110 min. Additionally, in the Danish multicenter study published in 2024, the mean operative time was 60 to 90 min [4, 5].

Regarding early postoperative complications (e.g., bleeding or leakage), there was one early postoperative complication in the form of leakage (10%). This case underwent reversal of RYGB due to chronic abdominal pain. On postoperative day 5, we noticed vital instability and repeated vomiting. After resuscitation, we requested a CT scan, which revealed leakage from the anastomotic line between the distal biliopancreatic limb and the proximal Roux limb, which was taken down and re-anastomosed. The patient experienced a smooth postoperative recovery and was discharged uneventfully 6 days later.

Our results are concordant with several published series, which report leak rates ranging from 5 to 10% following the reversal of bariatric surgery. For example, Snyder et al. conducted a multicenter review of 92 patients who underwent reversal of Roux-en-Y gastric bypass and reported a postoperative leak rate of 8.6% [10]. Similarly, Moon et al., using data from the MBSAQIP database, found a leak rate of 6.2% in revisional bariatric surgeries overall, highlighting the increased risk associated with these complex procedures [11].

Additionally, Pearl Ma et al. reported a leak rate of 10% in their study, describing a ninefold increase in leakage rate and nearly doubling the hospital stay following revisional procedures [12].

The current study reported one mortality due to liver cell failure. This patient has undergone OAGB 3 years prior to the presentation and suffered from severe malnutrition and liver cell failure. The patient received supportive treatment in the form of IV fluids, albumin, vitamins (thiamin, B12), electrolyte correction, and TPN. The patient was discharged after 1 week for a home regimen of TPN. After the MDT consultation, the decision was Made to proceed with a reversal. The patient was admitted to the ICU, and supportive treatment was administered to improve his general condition. Reversal was done after 5 days, but unfortunately, the liver cell failure led to generalized anasarca, and the patient died eventually.

This is comparable to the 15–20% morbidity rates reported in larger series (e.g., [10, 11]). Also, Pearl Ma et al. reported a mortality rate of 6% in a 3-year postoperative period [12].

Unlike Himpens et al., who reported no mortalities, our 10% mortality may reflect the inclusion of higher-risk patients with end-organ damage [1].

These findings address the technical demands of reversal surgery and suggest that lower complication rates need procedural standardization, surgeon experience, and multidisciplinary perioperative management.

The main indication for reversal in this pilot study was malnutrition (33%). In most cases, malnutrition could be managed conservatively by oral feeding, tube feeding, and/or TPN administration. However, when malnutrition becomes severe and resistant to standard treatment, the options for available care remain limited. Nutritional counseling serves as the primary intervention; however, in certain situations, surgical intervention—such as shortening the biliopancreatic limb and extending the common channel—may be considered. When dietary modifications alone cannot manage malnutrition and temporary nutritional support, such as total parenteral nutrition (TPN) or gastrostomy tube feeding, has been exhausted, a permanent surgical solution should be considered.

In our study, the mean albumin level preoperatively was 2.95, reflecting a moderate to severe protein depletion, while in other studies like Ahuja et al. [2], it has a reported value of 2.7 and a reported value of 2.9 in a study published by Sanchez-Pernaute et al. [2, 3].

The mean increase in albumin levels 6 months postoperatively was 0.6, which is comparable to other studies that range between 0.5 and 0.8 [1, 5]. These results are consistent with prior research highlighting the role of anatomical restoration in normalizing nutrient absorption.

Twenty-nine percent of our patients underwent reversal for excessive weight loss and inability to gain weight with nutritional counseling and supplementation.

A significant proportion of our patients (62.5%) experienced weight regain at 6 months post-reversal, with a statistically significant increase in BMI (*p* = 0.013). This aligns with findings from other studies, such as those by Ahuja et al. [2], where weight regain was reported in 50–70% of patients after reversal [2]. Additionally, our study demonstrated a significant improvement in albumin levels (*p* = 0.019), highlighting the effectiveness of reversal in addressing malnutrition.

A refractory marginal ulcer was an indication in one patient in our study. In most cases, a marginal ulcer can be managed conservatively with a stepwise approach, starting with lifestyle modification and oral proton pump inhibitor (PPI) administration, followed by intravenous PPI administration and then IV infusion. If failed or complicated, trials of endoscopic treatment can be tried. When marginal ulcers are refractory to all the previously mentioned measures, surgical intervention should be considered [12].

The patient’s symptoms were severe abdominal pain and bleeding, and the patient underwent several endoscopic trials but failed. His symptoms improved dramatically after reversal of RYGB, as evident by the questionnaire from.

This is aligned with a study published in 2025 stating that Marginal ulcers represented 14% of the main indications for gastric bypass reversals [13].

On the contrary, refractory marginal ulcer was reported to be the main indication for reversal (52%). Marginal ulcer is a frequent (> 10%) complication of RYGB, most commonly caused by *Helicobacter pylori *infection, tobacco use, or non-steroidal anti-inflammatory drugs [12]. All of these factors can be prevented or treated medically. That is why the selection of patients and the MDT approach in decision-making can be a factor in limiting the percentage of patients with marginal ulcers undergoing reversal procedures.

In our cohort, 75% of patients reported improved quality of life post-reversal, which is comparable to rates reported in other studies (e.g., 70–80% in [4]). However, 12.5% of our patients experienced worsening reflux, a known risk of reversal surgery. This highlights the importance of thorough preoperative counseling [4].

Our study provides detailed data on the laparoscopic reversal of both Roux-en-Y gastric bypass (RYGB) and one-anastomosis gastric bypass (OAGB). In contrast, most studies have focused solely on RYGB (e.g., [14]).

The inclusion of OAGB reversal addresses a gap highlighted in Robert et al.’s [15] systematic review, which reports a 21.4% higher risk of malnutrition in OAGB patients compared to RYGB patients [15]. The use of a standardized questionnaire to assess symptom severity and quality of life pre- and postoperatively enhances the objectivity of our findings. The statistically significant improvements in BMI and albumin levels provide robust evidence for the metabolic benefits of reversal in selected patients.

## Limitations

- The single-center nature of the study may limit generalizability.
- The small sample size (*n* = 10) limits the validity of our findings. Larger, multicenter studies are needed to validate these results.
- The short follow-up interval limits the effect of weight regain.
- The retrospective design introduces potential bias, such as incomplete data or recall bias. Prospective studies with longer follow-up would strengthen the evidence.

## Clinical Implications

The preliminary data from the current pilot study suggest the potential efficacy of laparoscopic reversal of gastric bypass for patients with severe, treatment-resistant complications; however, it should be reserved as a final resort when all conservative approaches have failed.

## Conclusion

Laparoscopic reversal of gastric bypass is a complex surgery requiring a specialized bariatric center and an experienced surgical team in revisional bariatric surgery. This kind of surgery should be a last resort for severe, intractable, chronic symptoms. Patient education about relatively high morbidity and the possibility of dissatisfaction is crucial.

## Data Availability

No datasets were generated or analysed during the current study.
